# Q-Der: a next-generation CoQ10 analogue supercharging neuroprotection by combating oxidative stress and enhancing mitochondrial function

**DOI:** 10.3389/fmolb.2025.1525103

**Published:** 2025-02-25

**Authors:** Matteo Micucci, Federico Gianfanti, Sabrina Donati Zeppa, Giosuè Annibalini, Barbara Canonico, Fabiana Fanelli, Roberta Saltarelli, Riham Osman, Mariele Montanari, Daniele Lopez, Gemma Nasoni, Giovanna Panza, Erik Bargagni, Francesca Luchetti, Michele Retini, Michele Mari, Giovanni Zappia, Vilberto Stocchi, Alessia Bartolacci, Sabrina Burattini, Michela Battistelli

**Affiliations:** ^1^ Department of Biomolecular Sciences, University of Urbino Carlo Bo, Urbino, Italy; ^2^ Department of Human Sciences and Promotion of the Quality of Life, University of San Raffaele, Roma, Italy; ^3^ Umolsystem Srl, Tecnopolo Roma-Castel Romano, Roma, Italy

**Keywords:** coenzyme Q10, Q-Der, oxidative stress, mitochondrial dysfunction, rotenone, neuroprotection, HT22 cells, ATP synthesis

## Abstract

**Background:**

Mitochondrial dysfunction and oxidative stress are central mechanisms in the progression of neurodegenerative diseases. This study first evaluated the toxicity of Q-Der (Q10-diacetate), a derivative of Coenzyme Q10, in HT22 hippocampal neurons under normal and oxidative stress conditions.

**Methods:**

HT22 cells were treated with Q-Der at 2.5, 5 and 10 µM with and without rotenone. Mitochondrial superoxide production (Mitosox), gene expression (via qRT-PCR), and protein levels (via Western blot) were measured. Morphological analyses were performed using transmission (TEM) and scanning (SEM) electron microscopes.

**Results:**

Q-Der significantly reduced mitochondrial superoxide levels, particularly at 5 μM, and upregulated key mitochondrial biogenesis genes, including PGC-1α and TFAM. Additionally, it restored the expression of MT-ND1 and MT-COI, which were downregulated by rotenone. Western blot results showed a significant recovery in CV-ATP5A (complex V) expression (p < 0.05), preserving mitochondrial ATP production. Morphological analyses further confirmed Q-Der’s ability to maintain cellular and mitochondrial structure under stress conditions.

**Conclusion:**

These findings suggest that Q-Der is non-toxic under normal conditions and protects against oxidative stress, supporting its potential as a therapeutic agent for neurodegenerative diseases.

## 1 Introduction

Neurodegeneration is an inflammatory response in the central nervous system (CNS). It represents a complex constellation of conditions characterized by the progressive decline in neurons’ structure, function, and viability. This pathological phenomenon is central to various disorders that collectively are known as neurodegenerative diseases (NDs). These conditions—such as Alzheimer’s disease (AD), Parkinson’s disease (PD), amyotrophic lateral sclerosis (ALS), Huntington’s disease (HD), and multiple sclerosis (MS)—are unified by their insidious onset and the inexorable deterioration of cognitive, motor, or sensory functions they inflict upon individuals.

These diseases share a gradual onset and a relentless progression of cognitive, motor, or sensory impairments, making them a leading cause of disability worldwide, particularly among older populations. As society ages, the prevalence of NDs is expected to increase, highlighting the critical need for enhanced understanding and development of innovative preventive strategies.

At the cellular level, neurodegeneration is characterised by a series of events including synaptic dysfunction, neuronal loss, and brain atrophy. These processes are driven by the neurons’ inability to sustain necessary biochemical and cellular operations, leading to apoptosis or other forms of cell death (Granatiero et al., 2019; Angelova and Abramov, 2018; Wilson et al., 2023). This results in the breakdown of neural networks and neurotransmission disruption, manifesting in symptoms depending on the affected brain regions. The molecular mechanisms at play, such as mitochondrial dysfunction, oxidative stress, and low-grade inflammation, are pivotal to understanding the pathology of NDs. Mitochondrial dysfunction compromises cellular energy metabolism, contributing to the cell death seen in NDs. Oxidative stress, resulting from an imbalance between reactive oxygen species (ROS) production and antioxidant defenses, further damages neurons (Lin and Beal, 2006). Key molecular players in these processes include oxidative stress induction and molecular networks related to neuroinflammation and cellular dysfunction, exacerbating neuronal damage (Sivandzade et al., 2019).

The environmental and lifestyle factors, including diet, physical activity, and exposure to toxins, further modulate the risk and progression of neurodegenerative diseases. Here, the role of diet emerges as both a potential risk factor and a protective ally against neurodegeneration (Solfrizzi et al., 2017; Zhang et al., 2021; Bianchi et al., 2021; Yang et al., 2021; Godos et al., 2024). Nutritional patterns rich in polyphenols and healthy fats can mitigate the risk of neurodegenerative diseases by influencing the molecular mechanisms of neurodegeneration. Diets that reduce oxidative stress, dampen low-grade inflammation, and improve mitochondrial function can significantly impact the progression of these diseases. Additionally, consistent consumption of fruits like berries and cherries, which are high in polyphenols, may contribute to maintaining cognitive health (Wang et al., 2023). Investigating phytocomplexes used in folk medicine could lead to the discovery of compounds with diverse preventive benefits across various diseases, including cancer (Micucci et al., 2024), cardiovascular issues (Micucci et al., 2020), and neurological disorders (Lapi et al., 2020).

The exploration of phytocomplexes derived from plants such as Acacia catechu (Chiaino et al., 2021), Castanea sativa (Brizi et al., 2016), Olea europea (Chiaino et al., 2020), and phytochemicals like flavonoids and curcumin have unveiled their potential to modulate critical molecular pathways involved in neurodegeneration. These substances can inhibit the activation of NF-κB, thereby reducing the production of pro-inflammatory cytokines such as IL-1 and IL-6. They also promote the activation of Nrf2, enhancing the cellular antioxidant response and protecting against oxidative damage (Abrahams et al., 2019; Calis et al., 2020; Farzaei et al., 2019). Indeed, generally, flavonoids lead to a reduction in ROS, regardless of their source (endogenous: mitochondria, peroxisomes, xanthine oxidase, Fenton reaction, NADPH oxidase, lipoxygenases, cytochrome P450 or exogenous: visible, UV and ionising radiation, chemotherapeutics) (Kicinska and Jarmuszkiewicz, 2020). Additionally, compounds naturally occurring in the human body, such as melatonin and Coenzyme Q10 (CoQ10), have also been studied and extracted, leading to the development of novel agents to mitigate oxidative stress and support mitochondrial function in neurodegenerative conditions (Areti et al., 2017; Bagheri et al., 2023).

Amidst these complex interactions, CoQ10 and its derivatives stand out for their dual role in mitochondrial function and antioxidant defence. Coenzyme q10 provides a promising avenue for neurodegerative disorders, acting on oxidative phosphorylation, which occurs in the mitochondria via the electron transport chain, the primary process responsible for ATP production (Kang et al., 2020). However, challenges in bioavailability and optimal dosing underscore the need for further research to harness CoQ10’s full potential in combating NDs.

This study introduces a pioneering *in vitro* comparison of a novel CoQ10 analog with CoQ10, focusing on their neuroprotective effects and underlying mechanisms in HT22 neuronal cells. Our investigation delves into mitochondrial ROS modulation, regulation of genes implicated in mitochondrial biogenesis and function (*PGC-1ɑ*, *TFAM*, *MT-ND1, MT-COI, MT-COX5B*), and OXPHOS subunit protein expression, aiming to illuminate the novel analog’s superior efficacy and mechanism profiles. Finally, SEM and TEM analyses suggest neuroprotective effects evidence by preserved mitochondrial integrity, reduced apoptotic cells, autophagic vacuoles, and cellular morphology in the treatment group compared to controls. This work represents a significant stride in overcoming CoQ10’s dosing challenges, potentially setting a new benchmark in neuroprotective strategies.

## 2 Materials and methods

### 2.1 Chemicals and reagents

CoQ10 was obtained from Sigma-Aldrich (C9538). CoQ10 derivative (Q-Der) was kindly provided by Umolsystem Srl. Rotenone was used as the neurotoxin at a concentration of 5 µM. Dimethyl sulfoxide (DMSO) was used as a vehicle control. Other reagents included fetal bovine serum (FBS), L-glutamine (100 mM), and antibiotics (penicillin and streptomycin). All chemicals were sourced from Sigma-Aldrich and prepared according to the manufacturer’s instructions.

### 2.2 Cell cultures

Mouse hippocampal neuronal cell line (HT22) was maintained in DMEM-Ham’s F12, supplemented with 10% fetal bovine serum, L-glutamine (100 mM), and 1% antibiotics (penicillin, streptomycin), and incubated in humidified 5% CO_2_ atmosphere at 37°C. At 80% confluence, cells were detached with trypsin-EDTA, washed, and sub cultivated in new flasks for 1–2 days before the experimental procedures. Neuroprotective effects were evaluated by pre-incubating cells with varying concentrations of Q10 and Q-Der for 3 or 24 h, followed by a 24-h exposure to 5 µM rotenone to induce neurotoxicity.

### 2.3 Experimental design

**FIGURE 1:**
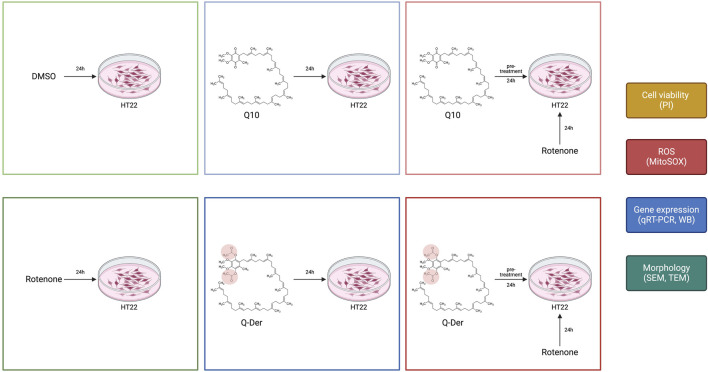
Design of the experimental procedure on HT22 cells in the different experimental conditions.

Cells were divided into six experimental groups, as reported in Figure 1:

− Control: cells treated with the vehicle (DMSO).
− Q10: cells treated with 5 µM Q10 for 24 h.
− Q-Der Low, Medium, High: cells treated with 2.5, 5, or 10 µM Q-Der for 24 h.
− Rotenone: cells exposed to 5 µM rotenone for 24 h to induce neurotoxicity.
− Q10 + rotenone: cells pre-treated with 5 µM Q10 for 3 or 24 h, followed by 24-h rotenone exposure.
− Q-Der + rotenone: cells pre-treated with 5 µM Q-Der for 3 or 24 h, followed by 24-h rotenone exposure.

### 2.4 Cell viability assay (Propidium iodide staining)

Propidium Iodide (PI) staining was performed to assess cell viability. PI is a fluorescent dye that penetrates only cells with compromised membranes, indicating cell death. Control and rotenone-challenged cells treated with 2.5, 5, and 10 µM Q-Der at 24 h post-treatment, were incubated with PI for 10 minutes. PI fluorescence was measured using a flow cytometer (FACSCanto II). The percentage of PI-positive cells, representing dead or necrotic cells, was calculated for each experimental group.

### 2.5 Mitochondrial ROS measurement (MitoSOX red assay)

Mitochondrial ROS production was measured using MitoSOX Red (Thermo Fischer, M36008), a dye specifically oxidized by mitochondrial superoxide. Control and rotenone-challenged HT22 cells treated with 2.5, 5, and 10 µM Q-Der for 24 h were incubated with 5 µM MitoSOX Red for 10 min at 37°C. Flow cytometry measured fluorescence intensity (excitation wavelength 561 nm, emission wavelength 610 nm). The results were expressed as percentages of MitoSOX positive cells.

### 2.6 qRT-PCR analysis

Total RNA was isolated from treated and control cells using the Omega Bio-Tek E.Z.N.A.™ Total RNA Kit following the manufacturer’s protocol. RNA concentrations were measured using the SpectraMax® QuickDrop™ Micro-Volume Spectrophotometer. cDNA was synthesized from 500 ng of total RNA using the Takara PrimeScript™ RT Master Mix. Quantitative real-time PCR (qRT-PCR) was performed using the StepOnePlus™ Real-Time PCR System (Applied Biosystems) and PowerUp SYBR Green Master Mix. Primers used for PGC-1α, TFAM, MT-ND1, MT-COI, COX5B, and S16 were designed based on previous studies (see Table 1 for primer sequences). Relative gene expression was normalized to S16 as the internal control, and data were analyzed using the 2^−ΔΔCT^ method.

**TABLE 1:** Primers used in real-time RT-PCR quantification.

Gene	Primer forward (5′-3′)	Primer reverse (5′-3′)
*PGC-1α*	ACTGAGCTACCCTTGGGATG	TAAGGATTTCGGTGGTGACA
*TFAM*	GGGAGCTACCAGAAGCAGAA	CTTTGTATGCTTTCCACTCAGC
*MT-ND1*	AGGCCCTAACATTGTTGGTCC	TGTGAGTGATAGGGTAGGTGC
*MT-COI*	TCTACTATTCGGAGCCTGAGC	CAAAAGCATGGGCAGTTACG
*COX5B*	CCGTCCATCAGCAACAAGAGAA	GCCAAAACCAGATGACAGTACAGT
*S16*	TGAAGGGTGGTGGACATGTG	ATAAGCTACCAGGGCCTTTGA

Note: *PGC-1α*, Peroxisome Proliferator-Activated Receptor Gamma Coactivator 1-alpha; *TFAM*, Transcription Factor A, mitochondrial; *MT-ND1*, Mitochondrially Encoded NADH: Ubiquinone Oxidoreductase Core Subunit 1; *MT-COI*, Cytochrome C Oxidase Subunit I; *COX5B*, Cytochrome C Oxidase Subunit 5B; *S16*, Mitochondrial Ribosomal Protein S16.

The target genes and the corresponding primer sequences used in qRT-PCR quantification are provided in Table 1.

### 2.7 Western blot analysis

Total protein extracts were obtained from the organic phase following the QIAzol protocol, solubilised in ISOT lysis buffer containing 8 M urea, 4% CHAPS, 65 mM DTE, 40 mM Tris base, 1 mM NaF, 1 mM Na_3_VO_4_, 1× complete protease inhibitor cocktail (Roche Diagnostics), then sonicated three times for 5 s on ice. After centrifugation at 12,000xg for 10 min, protein concentration was determined using Bradford colorimetric assay (Bio-Rad Laboratories) (Bradford, 1976). Equal amounts (30 μg) of total proteins were resolved in precast stain-free 4%–15% SDS polyacrylamide gels (Bio-Rad Laboratories, 4568084) and electrotransferred to polyvinylidene difluoride (PVDF) membranes (Bio-Rad Laboratories, 1620177) using the Power Blotter System semi-dry transfer device (ThermoFisher Scientific). Western blot analyses were performed using total oxidative phosphorylation (OXPHOS) cocktail from Invitrogen (458099) diluted 1:1,000 to detect individual complexes of the electron transport chain: CI-NDUFB8 (20 kDa), CII-SDHB (30 kDa), CIII-UQCRC2 (48 kDa), CIV-MTCO1 (40 kDa) and CV-ATP5A (55 kDa) (Welinder and Ekblad, 2011).

### 2.8 Morphological analyses environmental scanning electron microscopy (ESEM) and transmission electron microscopy (TEM)

Control and treated HT22 hippocampal cells were directly processed on coverslips in Petri dishes. After careful washing with 0.1 M phosphate buffer, monolayers were fixed with 2.5% glutaraldehyde in the same buffer for 1 h. All the specimens were post-fixed with 1% OsO_4_ in 0.1 M phosphate buffer for 1 h. After alcohol dehydration, they were critical point dried, gold-sputtered, and observed with an ESEM scanning electron microscope (UMKC, Kansas City, MO, United States). For TEM analysis, HT22 hippocampal cells, growing adherent in flasks, were washed, immediately fixed “*in situ*” with 2.5% glutaraldehyde in 0.1 M phosphate buffer for 30 min, gently scraped, and centrifuged at 1,200 rpm. The pellets were fixed in 2.5% glutaraldehyde for an additional 30 min. All the specimens were post-fixed in 1% OsO_4_ for 1 h, alcohol dehydrated, and embedded in araldite (Salucci et al., 2015). Ultrathin sections of the embedded samples were cut using a Leica UC7 ultramicrotome, and thin sections were stained with uranyl acetate and lead citrate and then analyzed with a Philips CM10 transmission electron microscope.

### 2.9 Comprehensive statistical analysis

For a holistic analysis of data derived from various assays discussed—including cell viability, mitochondrial functionality, and qRT-PCR for *PGC-1α*, *TFAM*, *MT-ND1*, *MT-COI*, and *COX5B* expression—statistical evaluation was meticulously designed to ensure the accuracy and reliability of the findings. Initially, all data were subjected to the Shapiro-Wilk test to assess normality, a critical step in determining the appropriate statistical methods for analysis. For data conforming to a normal distribution, analysis of variance (ANOVA) was employed to compare the mean values across different experimental and control groups. Subsequent *post hoc* analysis, utilizing Tukey’s and Sidak’s tests, helped identify specific groups showing statistically significant differences. In contrast, for datasets not meeting normal distribution criteria, the Kruskal-Wallis test was applied as a non-parametric alternative to ANOVA for comparing means across groups. Dunn’s *post hoc* test was then used for detailed pairwise comparisons to pinpoint where significant differences lay. The statistical analyses were performed using advanced software tools like GraphPad Prism, ensuring high precision and reliability. The significance threshold was consistently set at p < 0.05 across all tests. Results were reported as mean ± SD for parametric data sets or median with interquartile range for non-parametric datasets, appropriately representing the data’s central tendency and dispersion, from at least three independent experiments. Statistical significance was indicated as p < 0.05 (*), p < 0.01 (**), and p < 0.001 (***).

## 3 Results

### 3.1 Cell viability assessment (Propidium iodide staining)

**FIGURE 2:**
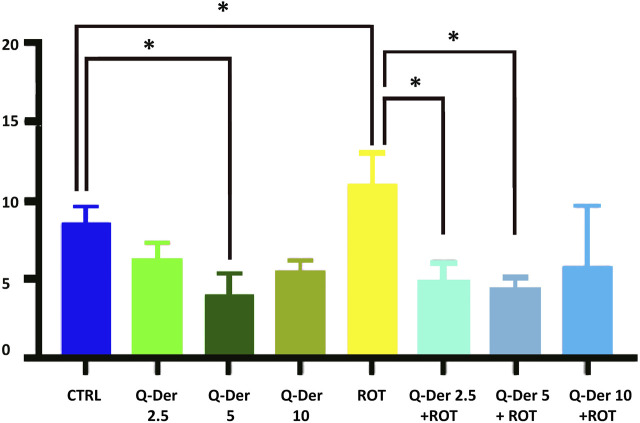
Q-Der effects on HT22 viability in healthy and rotenone-treated cells. The panel shows the percentage of PI positive cells, indicating cell death. Ordinary one-way ANOVA, Šidák’s multiple comparison, *P value <0.05, **P < 0.01, ***P < 0.001, ****P < 0.0001.

The results from the PI staining assay reveal distinct effects of Q-Der, both in the absence and presence of rotenone, on HT22 cell viability (Figure 2). Under baseline conditions, healthy cells treated with Q-Der at 5 µM show a reduction in PI-positive cells compared to the control. However, this decrease was not significant at 2.5 µM. These data suggest the *in vitro* safety profile of Q-Der under non-stressful conditions.

The neurotoxic effects of rotenone were evident, as indicated by a marked increase in PI-positive cells relative to the control. Pre-treatment with Q-Der at both concentrations significantly attenuates this rotenone-induced effect. Notably, the reduction in PI-positive cells in the Q-Der pre-treated groups approaches levels observed in control cells, indicating a robust protective effect against rotenone-mediated cytotoxicity.

These findings underscore the *in vitro* efficacy of Q-Der in preserving cell viability under oxidative stress conditions, with its protective effects becoming more pronounced in the presence of rotenone compared to the modest impact observed in the absence of the neurotoxin.

### 3.2 Mitochondrial superoxide production (MitoSOX red analysis)

**FIGURE 3:**
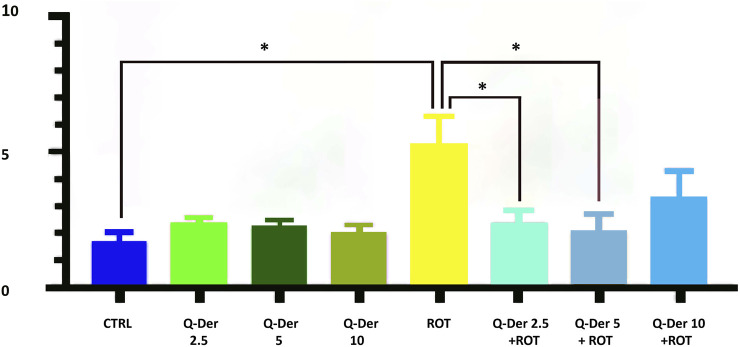
Evaluation of mitochondrial superoxide levels in control and rotenone-treated cells upon the treatment of 2.5, 5, and 10 µM Q-Der. Ordinary one-way ANOVA, Šidák’s multiple comparison, *P value <0.05, **P < 0.01, ***P < 0.001, ****P < 0.0001.

The results of the MitoSOX Red assay, used to quantify mitochondrial superoxide production, demonstrate the efficacy of Q-Der in mitigating oxidative stress induced by rotenone. In the control group, mitochondrial superoxide levels remain at baseline, suggesting physiological mitochondrial function. Mitochondrial ROS levels were not modified in healthy cells treated with Q-Der at all concentrations. On the contrary, rotenone-induced a substantial elevation in superoxide production. Pre-treatment with Q-Der at 2.5 µM and 5 µM reduced the rotenone-induced increase of mitochondrial ROS levels, nearly restoring them to baseline. Q-Der at 10 µM reduces mitochondrial superoxide levels to a lesser extent than the lower doses, suggesting a potential dose-dependent plateau. These findings indicate that Q-Der effectively counters oxidative stress at optimal concentrations, with a strong statistical significance in the protective effects observed at 2.5 µM and 5 µM. This underscores its potential to preserve mitochondrial superoxide levels under induced oxidative stress (Figure 3).

### 3.3 Gene expression analysis (qRT-PCR)

The qRT-PCR results reveal significant insights into the impact of Q-Der on the expression of key mitochondrial genes under basal and rotenone-induced conditions. The genes analyzed include *PGC-1α*, *TFAM*, *MT-ND1*, *MT-COI*, and *COX5B*, integral to mitochondrial biogenesis, respiratory function, and oxidative phosphorylation.

In the absence of rotenone, Q-Der at 5 µM induced a noticeable upregulation of *PGC-1α* expression, while CoQ10 at the same concentration did not affect this parameter. The expression of *TFAM*, a gene essential for mitochondrial DNA maintenance, was not modified by CoQ10 or Q-Der treatment. As expected, rotenone markedly reduced *TFAM* expression, both CoQ10 and Q-Der partially antagonized this effect. *MT-ND1* and *MT-COI*, which encode subunits of the mitochondrial respiratory chain complexes I and IV, showed a similar pattern: the active compounds partially restored their rotenone-decreased expression. Finally, *COX5B*, a nuclear-encoded Cytochrome C oxidase subunit (complex IV), increased its expression only in rotenone-challenged cells treated with CoQ10 and Q-Der, with Q-Der showing a higher potency.

**FIGURE 4:**
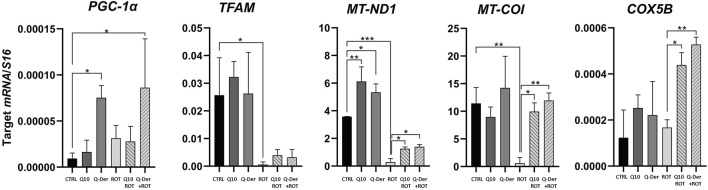
Mitochondrial-Related Gene expression. HT22 mRNA expression levels of *PGC-1ɑ*, *TFAM*, *MT-ND1*, *MT-COI* and *COX5B* in healthy and rotenone-treated cells challenged with CoQ10 and Q-Der at 5 μM *P < 0.05, **P < 0.01, ***P < 0.001.

The qRT-PCR analysis reveals that Q-Der is critical in promoting mitochondrial gene expression, particularly under oxidative stress conditions. The significant upregulation of PGC-1α, partial recovery of TFAM, and restoration of MT-ND1 and MT-COI expression highlight Q-Der’s efficacy in maintaining mitochondrial function and biogenesis (Figure 4).

### 3.4 Western blot analysis of OXPHOS complexes

**FIGURE 5:**
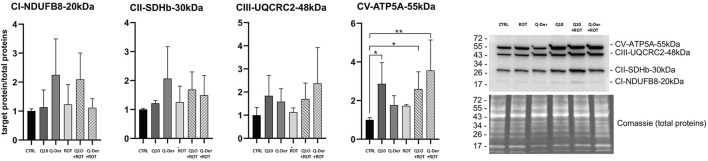
OXPHOS protein expression. Densitometric analyses of subunits of complexes I (CI-NDUFB8, 20 kDa), II (CII-SDHB, 30 kDa), III (CIII-UQCRC2, 48 kDa) and V (CV-ATP5A, 55 kDa) of the OXPHOS system normalised to total proteins. A representative Western blot image of OXPHOS and total proteins is shown on the right side of the figure.

The Western blot analysis of the OXPHOS complexes reveals key findings, particularly in relation to the expression of CI-NDUFB8 (complex I), CII-SDHB (complex II), CIII-UQCRC2 (complex III), and CV-ATP5A (complex V). Despite the range of experimental conditions, the analysis shows that the expression of CI-NDUFB8 remains unaffected by rotenone treatment. There is no statistically significant alteration in CI-NDUFB8 levels across all groups, including both Q-Der and CoQ10 pre-treatments. This stability indicates that rotenone does not exert a detectable impact on complex I subunit expression under these experimental conditions, and neither Q-Der nor Q10 significantly modifies CI-NDUFB8 levels. Similarly, the expression of CII-SDHB and CIII-UQCRC2 follows a comparable trend, with no statistical significant differences were observed between the control, rotenone, and pre-treatment groups. Although there is a slight trend toward recovery in the pre-treated groups, these changes do not meet the threshold for statistical significance. These observations suggest that while a minor biological effect may exist, it is not strong enough to be conclusive in terms of altering the expression of complex II and complex III subunits. The only subunit showing a statistically significant change is CV-ATP5A (complex V). Q-Der increased this subunit expression only in rotenone-treated cells, while CoQ10 determined a similar effect both in control and rotenone-treated cells (Figure 5).

### 3.5 Morphological analysis

#### 3.5.1 Scanning electron microscopy observation

**FIGURE 6:**
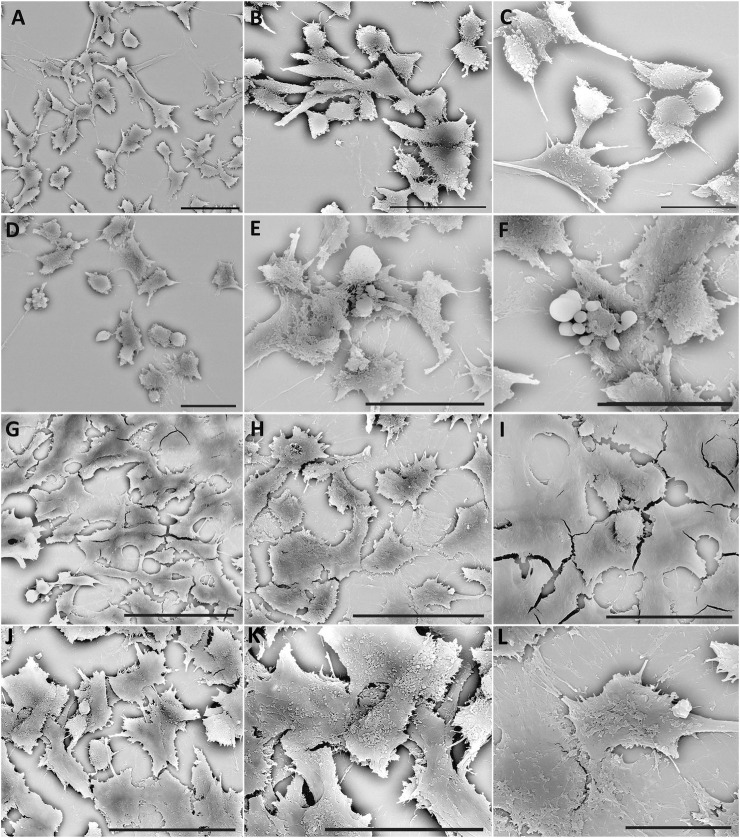
SEM of HT22 hippocampal cells. Control cells **(A–C)**; cells treated with rotenone (5 µM) for 24 h **(D–F)**; rotenone-treated cells pretreated with Q10 (5 µM) for 24 h **(G–I)**, rotenone-treated cells pretreated with Q-Der (5 µM) for 24 h **(J–L)**. **(A, B, D–I)**, Bar = 20 µm; **(C, J, K)**, Bar = 10 µm.

In the control condition at 24 h, cell morphology appears well preserved, HT22 cells appear elongated, and sometimes, some show a slightly rounded shape Figures 6A–C. The cells appear confluent and generate a carpet by forming extracellular membrane protrusions that create bridges between adjacent cells.

24 h of rotenone treatment induces a deep change in cell morphology; the HT22 appears predominantly rounded, probably due to a rearrangement of the cytoskeletal structure (Figures 6D–F).

The cells show fewer cytoplasmic protrusions, and a loss of extracellular junctions reduces cell carpet formation. Numerous blebs on the cellular membrane and the apoptotic bodies demonstrate the presence of numerous apoptotic cells.

After pre-treatment with Q10, the morphology is restored to the control’s; new cell junctions (Figures 6G–I) can be formed. Finally, pre-treatment with Q-Der shows the cells all flattened, more confluent, and with characteristic microvilli on the surface (Figures 6J–L). Both Q10 and Q-Der can reduce the percentage of apoptotic cells and induce the percentage of apoptotic cells and the preservation of cell communication.

#### 3.5.2 Transmission electron microscopy observation

**FIGURE 7:**
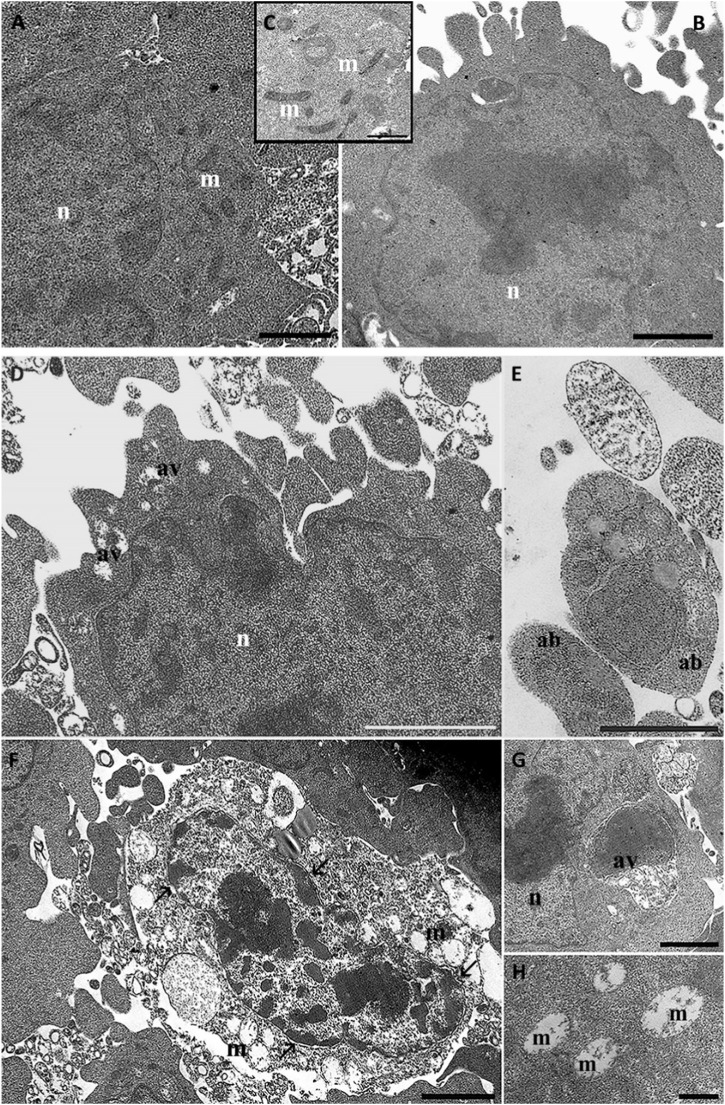
HT22 hippocampal cells at TEM. Control **(A, B)**, cells treated with rotenone (5 µM) for 24 h **(C–F, G)**. m: mitochondria; n: nucleus; ab: apoptotic bodies; av: autophagic vacuole; →: chromatin condensation. **(A, B)**, Bar = 1 μm; **(C, F)**, Bar = 500 nm; **(D, E)**, Bar = 2 µm.

For all experimental conditions we evaluate three grids. Every grid consisted of ten thin sections. Therefore, we performed the evaluation of the ultrastructural changes in 200–300 cells. The ultrastructural evaluation evidence good cell viability in control conditions (Figures 7A–C). HT22 cell morphology appeared elongated and confluent. After rotenone treatment, several rounded cells with blebs and apoptotic bodies appeared (Figures 7D–F). We can observe numerous autophagic vacuoles (G), with different sizes ranging from 200 to 500 nm. In the nucleus, we can observe chromatin condensation and a diffuse detachment of the nuclear membrane. Mitochondria appear swollen and heavily damaged; their maintained membrane is well preserved but appears well preserved while create are completely disrupted.

**FIGURE 8:**
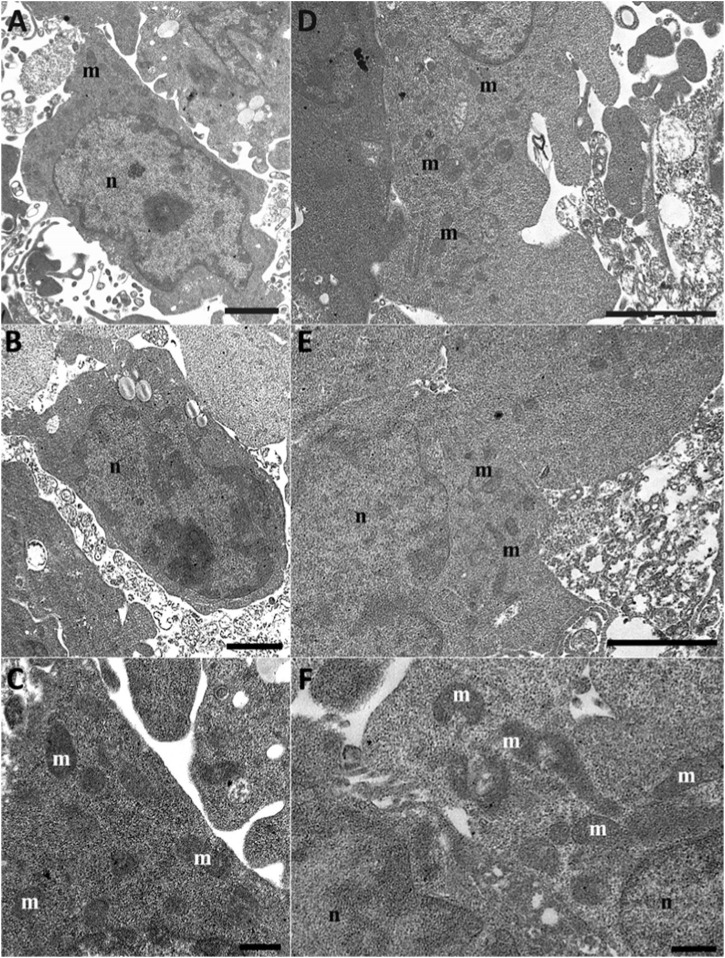
HT22 hippocampal cells at TEM. Rotenone-treated cells pretreated with Q10 (5 µM) for 24 h Q10 **(A–C)** and rotenone-treated cells pretreated with Q-Der (5 µM) for 24 h Q-der **(D–F)**. m: mitochondria; n: nucleus. **(A–C, E)**, Bar = 2 μm; **(C, F)**, Bar = 500 nm.

After Q10 treatment, the cells appeared confluent and showed well-preserved mitochondria (Figures 8A–C) comparable to the control conditions. A better mitochondria morphology appeared when cells were treated with Q10-der (Figures 8D–F). We can see well-preserved mitochondria that appear more numerous if compared pre-treatment to control or rotenone treatment. Mitochondria are present in hight number but smaller than control condition, suggesting a mitochondrial biogenesis.

## 4 Discussion

The findings from this study highlight the significant protective effects of Q-Der on HT22 neuronal cells, particularly under conditions of oxidative stress induced by rotenone. This condition offers a targeted model for studying mitochondrial dysfunction, as it selectively inhibits Complex I of the electron transport chain, disrupting ATP synthesis and causing localized ROS generation (Menke et al., 2003; Moon et al., 2005). This inhibition leads to mitochondrial membrane depolarization, increased expression of mitochondrial fission markers and activation of caspase-3-dependent apoptotic pathways. These effects are highly relevant to neurodegenerative diseases like Parkinson’s disease, where mitochondrial dysfunction and selective dopaminergic neuronal death are central features (Moon et al., 2005; Li et al., 2017). Several stressors act on neurons through the affection of the same pathways targeted by rotenone. For example, H₂O₂ induces oxidative stress generating ROS across multiple cellular compartments, leading to generalized oxidative damage that is not specific to mitochondria. Despite this distinction, both rotenone and H₂O₂ share some key effects, including ROS generation, mTOR pathway suppression, apoptotic activation involving caspase-3 cleavage and PARP fragmentation. These shared mechanisms provide complementary insights into oxidative stress and neuroprotection, with rotenone being a specific tool for the study of mitochondria-dependent neurodegenerative pathologies (Panee et al., 2007; Zhou et al., 2015; Giraldo-Berrio et al., 2024; Roy et al., 2023; Millichap et al., 2024).

Our study focuses on rotenone-induced oxidative stress due to its mitochondria-specific effects. Our results add to a growing body of evidence that underscores the importance of targeting mitochondrial dysfunction in neurodegenerative diseases such as Parkinson’s and Alzheimer’s, where oxidative stress plays a pivotal role. Q-Der demonstrated substantial efficacy in reducing cell death, restoring mitochondrial superoxide levels, and modulating key gene expressions involved in mitochondrial biogenesis and respiration. While the protective effects of Q10 have been well-documented in similar models (Bhagavan and Chopra, 2006), the current data suggest that Q-Der may offer superior benefits, particularly in maintaining mitochondrial homeostasis under stress conditions.

Chemically, Q-Der differs from Coenzyme Q10 in that it carries two acetyl groups on its quinone ring, increasing its lipophilicity and potentially enhancing its stability against oxidation. While CoQ10 functions through rapid cycling between oxidized and reduced forms to neutralize reactive oxygen species, the addition of acetate groups in Q-Der is theorized to confer a protective effect on the molecule itself, reducing its reactivity to oxidative agents. This structural modification may support a prolonged antioxidant presence within the mitochondrial membrane, thus maintaining cellular protection over extended periods of oxidative stress. These properties underscore Q-Der’s potential as a neuroprotective agent, particularly in environments where mitochondrial stability and sustained antioxidant effects are essential.

A key aspect of this study is the ability of Q-Der to preserve mitochondrial function and ROS levels. Compared to Q10, Q-Der consistently showed a trend toward better protection against oxidative stress markers such as mitochondrial superoxide, although this trend did not reach statistical significance. This mirrors findings in other studies where derivatives of Q10, such as MitoQ and Ubidecarenone, have been shown to exhibit enhanced mitochondrial targeting and antioxidant properties compared to the parent compound (Smith and Murphy, 2010). MitoQ, a mitochondria-targeted form of CoQ10, has demonstrated strong efficacy in reducing mitochondrial ROS and improving bioenergetics by selectively accumulating in the mitochondrial membrane, facilitating more effective protection against oxidative damage (Cochemé et al., 2007). Although MitoQ and Q-Der share similarities in their mechanisms of action—both aim to enhance mitochondrial function by modulating ROS levels—the differences in their molecular structures likely contribute to their varying degrees of efficacy. For instance, MitoQ’s triphenylphosphonium cation facilitates its direct accumulation in the mitochondria, while Q-Der may rely more on its metabolic conversion within cells.

The qRT-PCR analysis further underscores the potential of Q-Der, particularly in its ability to upregulate key genes involved in mitochondrial biogenesis, such as *PGC-1α* and *TFAM*. This is consistent with literature indicating that compounds enhancing *PGC-1α* activity could promote mitochondrial biogenesis and repair, thereby offering neuroprotection (Wenz, 2009). The upregulation of *PGC-1α* and partial rescue of *TFAM* under rotenone-induced oxidative stress suggest that Q-Der helps restore mitochondrial integrity and function, which is crucial for maintaining ATP production and preventing cellular apoptosis. Comparatively, studies on other Q10 derivatives like Q10-hydroxydecyl benzoate (IDE) have shown similar mitochondrial-protective effects, particularly in preventing the loss of complex I and complex III activity, further highlighting the importance of preserving mitochondrial bioenergetics in disease models (Villalaín, 2004). However, Q-Der’s ability to modulate both mitochondrial biogenesis and complex V expression distinguishes it as a promising candidate for further exploration.

The Western blot analysis revealed that while there were no significant changes in the expression of CI-NDUFB8, CII-SDHB, or CIII-UQCRC2 across the treatment groups, Q-Der significantly increased CV-ATP5A (complex V) expression in rotenone-treated cells. This result is particularly noteworthy, as complex V is essential for ATP synthesis, and its dysfunction is a critical contributor to energy deficits observed in neurodegenerative diseases (Nicholls and Budd, 2000). The statistically significant recovery of CV-ATP5A in Q-Der-treated cells indicates that this novel compound effectively preserves mitochondrial ATP production capacity under oxidative stress conditions. This protective effect on ATP synthase has also been observed in studies using idebenone, a synthetic analog of CoQ10, which has shown promise in clinical trials for conditions such as Friedreich’s ataxia and Leber’s hereditary optic neuropathy (Schiff and Rustin, 2016; Lyseng-Williamson, 2016). While idebenone primarily targets complex I deficiency, the broader effect of Q-Der on many mitochondrial parameters suggests a more comprehensive mitochondrial support mechanism.

An interesting finding in our study is that, despite QDer improving numerous parameters of mitochondrial function, it does not appear to increase the levels of UQCRC2, a key component of Complex III of the electron transport chain. This result may seem to contrast with well-established CoQ10-mediated regulation of other mitochondrial markers (Yousef et al., 2019). One possible explanation is that QDer exerts its effects primarily on Complex I or V, leaving Complex III relatively unaffected. Additionally, UQCRC2 levels might already be saturated under basal conditions, leaving little room for further regulation. Studies on PARL-deficient models demonstrate selective defects in Complex III mediated by TTC19 instability without impacting other complexes of the ETC (Spinazzi et al., 2019).

Furthermore, the partial increase in Complex III activity observed in idebenone-treated models suggests that increased protein expression does not always correlate with functional recovery (Llewellyn et al., 2015). Similarly, the unchanged GSSG:GSH ratio in the cerebellum suggests that certain oxidative stress markers may resist modulation even under treatment.

Morphologically, the structural preservation observed through SEM and TEM further supports the molecular data. Even after rotenone exposure, cells pre-treated with Q-Der exhibited well-preserved cellular morphology and mitochondrial structure. This contrasts with the significant cytoskeletal and mitochondrial damage seen in rotenone-treated cells, aligning with the concept that maintaining mitochondrial integrity is crucial for cellular survival under oxidative stress. Studies on other derivatives, such as SkQ1 (a plastoquinone derivative), have also demonstrated the importance of targeting mitochondrial health to prevent structural damage in neurons, supporting the idea that mitochondrial-targeted therapies can significantly affect disease progression (Fedorov et al., 2022; Sacks et al., 2021). However, while SkQ1 is known for its anti-apoptotic properties, Q-Der offers a broader protective mechanism by reducing oxidative stress and enhancing mitochondrial gene expression, further solidifying its role as a potential neuroprotective agent.

In conclusion, the results of this study place Q-Der in a promising position as a neuroprotective agent aimed at preserving mitochondrial function, particularly in NDs. While other derivatives of Q10, such as MitoQ, idebenone, and SkQ1, have shown efficacy in specific mitochondrial pathways, Q-Der appears to provide a multifaceted approach, combining antioxidant effects, mitochondrial biogenesis and enhanced ATP production. The statistical significance observed in the restoration of CV-ATP5A expression and the trends toward improvement in mitochondrial superoxide levels and gene expression positions Q-Der as a strong candidate for further *in vivo* studies and eventual clinical translation. Future research should aim to explore the exact molecular pathways through which Q-Der exerts its effects and to compare its efficacy *in vivo* against other Q10 derivatives in models of neurodegeneration. Additionally, a deeper understanding of the pharmacokinetics and bioavailability of Q-Der relative to other mitochondrial-targeted therapies will be crucial in determining its therapeutic potential.

## 5 Conclusion

The present study provides compelling evidence of Q-Der’s neuroprotective efficacy in HT22 cells, highlighting its ability to attenuate rotenone-induced oxidative stress and preserve mitochondrial function. Q-Der’s effects were evident through significant reductions in mitochondrial superoxide production, enhanced expression of mitochondrial biogenesis genes (PGC-1α and TFAM), and the upregulation of complex V ATP synthase, contributing to sustained ATP synthesis under stress conditions. These findings support Q-Der as a possible candidate for further investigation in neurodegenerative disease models, particularly those characterized by mitochondrial dysfunction and oxidative stress.

While this study establishes Q-Der’s protective effects *in vitro*, its translational potential requires further examination. The exclusive use of HT22 cells limits applicability across broader neuronal models, and *in vivo* studies are essential to confirm bioavailability and metabolic stability. Future research should expand to diverse cellular models and focus on Q-Der’s pharmacokinetics to substantiate its therapeutic promise.

## Data Availability

The raw data supporting the conclusions of this article will be made available by the authors, without undue reservation.
