# Supplementary figures and images for: Q-Der: a next-generation CoQ10 analogue supercharging neuroprotection by combating oxidative stress and enhancing mitochondrial function
Source: Front Mol Biosci. 2025 Feb 25;12:1525103. doi: 10.3389/fmolb.2025.1525103 (PMC11893404; doi:10.3389/fmolb.2025.1525103)

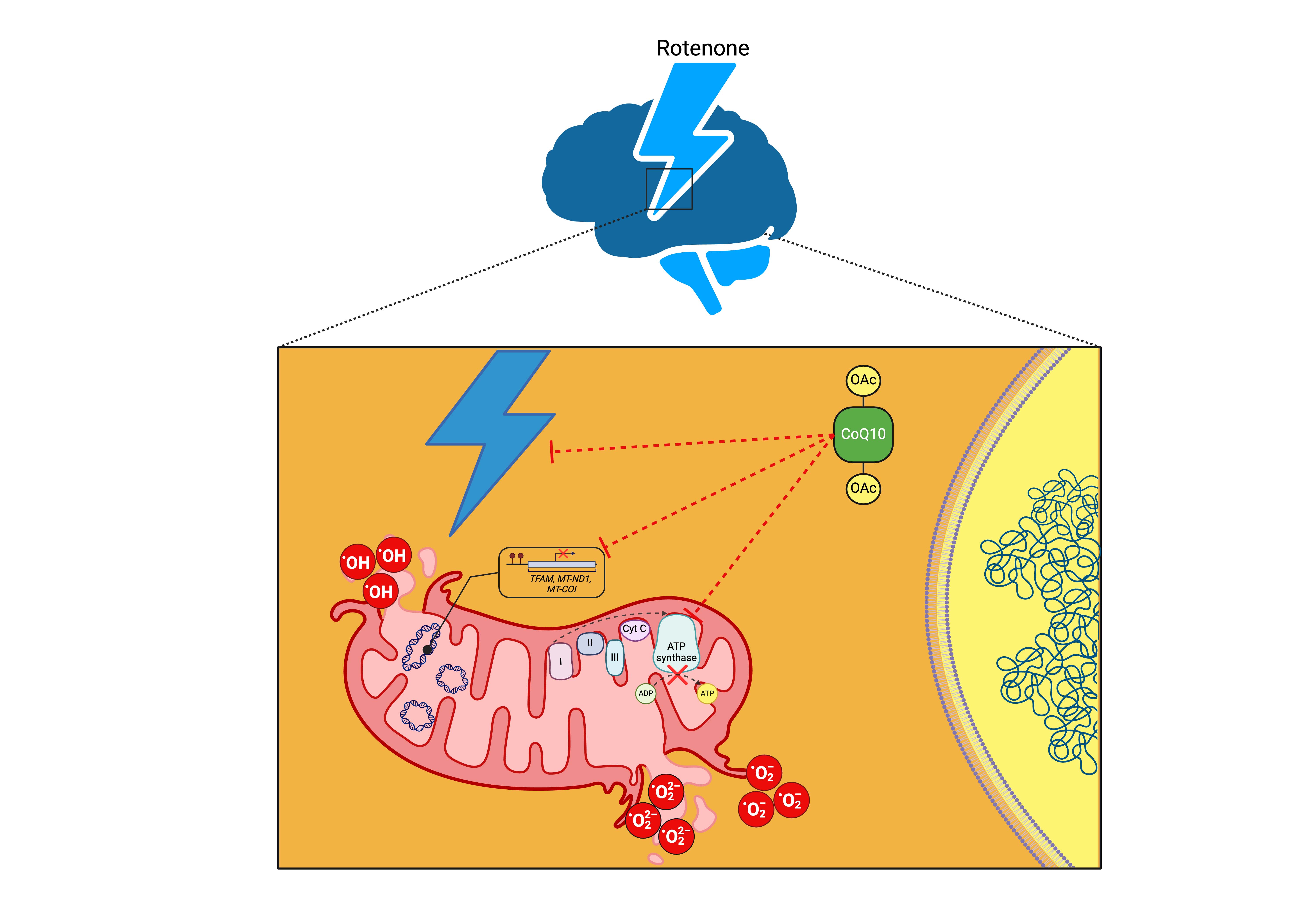

Supplement: Supplementary file 1 [file Image1.jpeg]
